# Diagnosis of Multiple Faults in Rotating Machinery Using Ensemble Learning

**DOI:** 10.3390/s23021005

**Published:** 2023-01-15

**Authors:** Udeme Ibanga Inyang, Ivan Petrunin, Ian Jennions

**Affiliations:** 1 Integrated Vehicle Health Management Centre, Cranfield University, Cranfield MK43 0AL, UK; 2 Centre for Autonomous and Cyberphysical Systems, Cranfield University, Cranfield MK43 0AL, UK

**Keywords:** comprehensive, multiple faults, gear, bearing, shaft, optimization, scales

## Abstract

Fault diagnosis of rotating machines is an important task to prevent machinery downtime, and provide verifiable support for condition-based maintenance (CBM) decision-making. Deep learning-enabled fault diagnosis operations have become increasingly popular because features are extracted and selected automatically. However, it is challenging for these models to give superior results with rotating machine components of different scales, single and multiple faults across different rotating components, diverse operating speeds, and diverse load conditions. To address these challenges, this paper proposes a comprehensive learning approach with optimized signal processing transforms for single as well as multiple faults diagnosis across dissimilar rotating machine components: gearbox, bearing, and shaft. The optimized bicoherence, spectral kurtosis and cyclic spectral coherence feature spaces, and deep blending ensemble learning are explored for multiple faults diagnosis of these components. The performance analysis of the proposed approach has been demonstrated through a single joint training of the entire framework on a compound dataset containing multiple faults derived from three public repositories. A comparison with the state-of-the-art approaches that used these datasets, shows that our method gives improved results with different components and faults with nominal retraining.

## 1. Introduction

Critical components in rotating machines in various industrial plants around the world include gears, bearings, and shafts. To ascertain the condition of this rotating machinery, vibration analysis—a non-invasive technique—is widely used due to its sensitivity to rotating-machine faults. Vibration analysis in condition monitoring ensures rotating machine faults can be detected on time, the location of such faults can be identified, and the future condition of the machine components can be predicted. With the rapid development in science and technology, artificial intelligence-based fault diagnosis methods utilized in various condition monitoring systems of rotating machines is vital to prevent financial losses, reduce unscheduled shutdown of the machine, prevent catastrophic failure, entrench the general safety of the staff, and protect the environment.

Artificial intelligent algorithms are adaptive, robust, and some do not require prior knowledge of the machine’s condition or parameters of the machine. The operation of an artificial intelligent-based fault diagnostic system can broadly be divided into data acquisition, fault feature extraction/feature selection and fault detection/identification [1]. The data acquisition involves the processes whereby data is collected and stored through the use of different types of sensors attached to the rotating machine to be monitored. Fault features obtained through the feature extraction algorithms are provided as inputs to the artificial intelligent tool from which pattern recognition is performed by mapping information obtained from feature space to the machine faults [2]. Artificial intelligence algorithms used in rotating machine diagnosis can be majorly grouped into shallow machine learning methods, deep learning methods, and transfer learning methods.

The shallow machine learning-based diagnostic systems involve fault feature extraction, where statistical features are obtained from manipulation of the raw vibration dataset [3], fault feature selection to improve the relevance between the features through methods such as Independent Component Analysis, and decision making. Although these methods give a fair performance for the fault diagnostic operations [4,5,6], they encounter problems in extracting useful information from large datasets, they are domain specific and require much human effort [7]. These problems affect the overall accuracy of such models and their flexibility across different rotating machine components operating in different conditions and environments.

On the other hand, deep learning algorithms, automatically carry out both the feature extraction/feature selection processes. Deep learning models require less human effort and knowledge of the system. They work well with a big, complex dataset, and help develop end-to-end systems [4]. Some examples of deep learning models include Convolutional Neural Networks, Autoencoders, and Deep Belief Networks.

The CNN is popular and widely used in machine fault diagnosis because of its good performance and ability to learn quickly [8]. Eren et al. [9] used the 1D CNN for a timely and accurate bearing diagnosis system. In a similar work, Eren et al. [10] evaluated the performance of a generic real-time fault diagnosis approach based on their developed 1D adaptive CNN model. Eren’s [9] and Hsiaso’s [11] results showed decent performance of the 1D CNN models. Souza et al. [12] tackled the financial cost associated with diagnostic systems using data from multiple sensors by developing a Predictive Maintenance model with a 1D Convolutional Neural Network (PdM-CNN) that operates on a vibration dataset from one sensor installed on the drive end of the test rig. Souza validated their model and obtained satisfactory results on the MaFaulDa and the Case Western Reserve University Datasets.

However, the 1D CNN learning from the raw vibration dataset suffers from shift invariance problems. Other methods such as adaptive overlapping 1D CNN have been proposed by Qian et al. [13] to deal with this challenge. Their solution deployed an adaptive convolutional layer and the root-mean-square (RMS) pooling layer in the 1D CNN system. To also address the issue of shift invariance and enhance fault features for easy detection, different signal processing transforms of the raw vibration data have been exploited as inputs to 2D CNN. Hence, Verstraete et al. [14] developed a diagnostic method based on 2D CNN and short time Fourier transform of the raw dataset for bearing fault diagnosis. Hoang et al. [15] integrated a multi-sensor fusion layer into a CNN with time-frequency wavelet inputs for bearing diagnosis. Images from a constant-Q transform were used by Pham et al. [16] as inputs to 2D CNN for compound fault diagnosis of bearing under varying operating speeds. Dibaj et al. [17] explored fine-tuned Variable Mode Decomposition (VMD) inputs to CNN for compound fault detection of gear and bearings with unequal severity. An overview of preprocessing methods such as fast Fourier transform, wavelet transform, s-transform, and cyclic spectral analysis used with CNN for rotating machine diagnosis was presented by Tang et al. [18]. Other researchers [19,20,21,22,23,24,25] have explored different signal-processed inputs to CNN for rotating machine diagnosis.

A key essence of signal processing is to highlight the fault characteristic features in the vibration signal for easy discrimination by the CNN. Although the different signal processing techniques have their intrinsic advantages, they also have some weaknesses. For instance, while the fast Fourier transform is fast, the FFT may generate scaling errors when the signal is non-stationary [26], and inputs based on this method may result in poor performance from the CNN if the bearing or gear signal has frequency components that are time-varying [25]. Similarly, the VMD is a powerful signal processing technique for decomposing a signal into different mode functions. The VMD requires that two of its parameters be set appropriately—the α penalty factor and the number of mode parameter *K*. However, setting these parameters without prior knowledge of the signal itself is a daunting task, and this affects the performance of the VMD when used independently and as an input preprocessing tool for CNN. On the other hand, the wavelet transform is suitable for analysing transient signals and it uses the basis function to match a specific fault condition [27]. However, the wavelet transform finds it challenging to split high frequency bands where modulation information of bearing and gear faults resides [28]. The kurtogram, a graphic representation of the spectral kurtosis, is another method that has been used to preprocess CNN inputs because of its ability to identify resonance frequency bands and extract the fault features [29]. Nonetheless, with fast spectrum kurtosis, in cases where strong, weak resonance bands and noise interference may be present, the weaker fault band may be challenging to identify [29].

Rotating machinery exists in diverse types and scales, and operates in different conditions of speed, varied load, and noise interference. However, from previous paragraphs, it was observed that signal processing transforms have different performance abilities under different conditions and equipment sizes. Hence, it is important to explore methods that would give a better performance from intelligent diagnostic systems across different conditions of operation and equipment size/types. An example of such a method is the selection of complementary signal processing techniques for such tasks.

Ensemble Learning—a machine learning method where two or more machine learning models are combined or fused—can be deployed to obtain better predictive performance. Ensemble learning methods such as boosting, bagging and decision fusion methods (for example, weighted averaging, majority voting, and maximum) are widely used and found to be effective in the area of rotating machine diagnostics and prognostics. When the training data is small, ensemble learning can also be advantageous in reducing the risk of choosing a wrong model by avoiding the local optimum, through the aggregation of all the individual models [30,31].

Xu et al. [32] addressed the problem of obtaining good results from bearing raw dataset by proposing a combination of a pre-trained CNN having greyscale images as input and random forest ensemble learning. Xu validated their model using two different datasets and obtained good accuracy. CNN, stacked Autoencoder, and Deep Belief Network formed an ensemble model proposed by Ma et al. [33] for end-to-end diagnosis of rotor and bearing faults. The whole essence of this combination was to overcome the adaptability problems of manually designed features in shallow learning models. Li et al. [34] proposed an ensemble of Convolutional Neural Network that takes inputs of root mean square maps from the FFT of two vibration sensors. Their results showed high diagnostic performance and adaptability on the Case Western Reserve University Bearing dataset. The influence of track excitation and other equipment’s vibration which may result in the presence of non-gaussian noise in the vibration signal was tackled by Jin et al. [35] through the use of multi-objective VMD optimization and ensemble learning for rotating machine diagnosis.

A framework that contains two 1D CNN branches and one multi-channel fusion CNN branch was combined with the SVM to address the challenges of information loss in sensor fusion for bearing fault detection [36]. Hence, coupling features were extracted from the multi-channel CNN and intrinsic features based on each of the sensors were extracted from the 1D CNN for bearing fault detection. Still addressing information loss, Li et al. [37] proposed an ensemble of CNN and DNN for bearing fault diagnosis. An ensemble of 1D CNN was explored by Hsiao et al. [11] to monitor transmission error of a worm gearbox. They also classified new, in-operation, and old conditions of such gears, and obtained good effectiveness. Other deep ensemble learning models in the literature were mostly focused on diagnosing and improving diagnostic results for single fault in rotating machines.

From the discussion above, ensemble deep learning models have helped address information/sensor fusion, improving predictive performance in varied conditions of machines, diagnostics in the presence of gaussian noise, and small dataset issues. However, research on multiple faults was minimal. Hence, the contributions of this article are as follows: to provide an implementation of blending ensemble-based fault diagnostics framework capable of diagnosing multiple faults from multiple components; parameter optimization for multiple components; and detailed performance analysis with outlined complementarity from different branches of the proposed framework. Hence, the proposed implementation is flexible, having the ability to give superior results with a small dataset, across diverse load and speed conditions of different rotating machine components of various scales.

The subsequent parts of this paper include Section 2, where the proposed architecture for implementation is presented, the theoretical explanation of selected signal processing techniques is proved, and the selected deep learning method and ensemble learning approach are explained. All the experimental setups and experimental description are highlighted in Section 3. The results from the various experiments are presented and discussed in Section 4, while Section 5 provides the conclusion to this article.

## 2. Method

**Figure 1:**
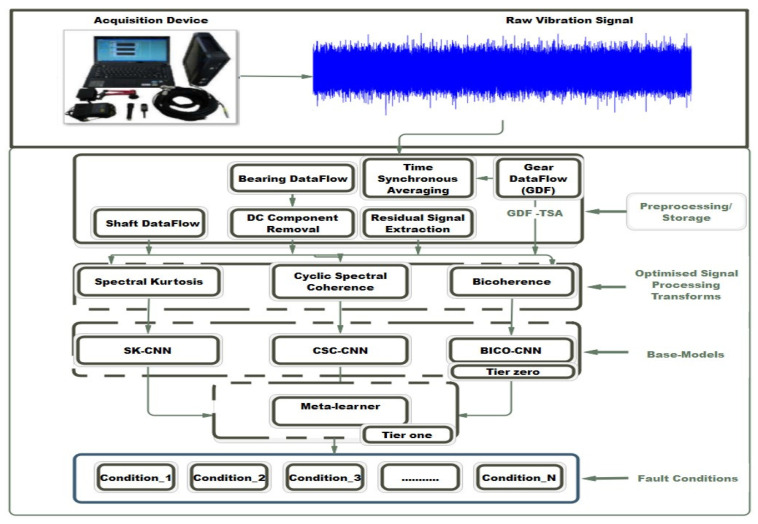
Proposed multiple faults diagnosis framework.

To solve the problems of lack of flexibility and scalability caused by a small dataset, varied load/different speed conditions, diverse types of rotating machine components, and single/multiple faults, the framework shown in Figure 1 was proposed for the diagnosis of rotating machine faults.

The major elements of the framework are divided into distinct phases that include the vibration signal acquisition using an accelerometer mounted on the principal rotating components. The next phase of the framework involves preprocessing of the acquired dataset; where operations such as DC component removal, Time Synchronous Averaging (*TSA*), and residual signal extraction are performed. The subsequent phase involved input creation using optimized parameters for the selected signal processing methods. Furthermore, the base learners, which are made up of three CNN models, form phase three of the framework. The base learners are homogenous, having a similar algorithm. Thus, preserving the simplicity of the framework. A trained meta-learner constitutes the last phase of the proposed framework. The Algorithm 1 is described in the steps below.
**Algorithm 1:** Blending ***Input:****Training Data*$D_{Train}={\left(x_{i},y_{i}\right)}_{i=1}^{m}$*, Optimized parameter of signal processing transform*$\;SP_{t1}^{1}$*….*$SP_{t+T}^{1}$*are found, tier zero learners*$CNN_{t1}^{1}$*….*$CNN_{t+T}^{1}$*and tier one meta learner.*
*Step 1: Train tier zero learners*
   *For*
$t_{1}$
*= 1, 2, …. T **do***

      *Apply Optimized*
$SP_{t1}^{1}$
*on*
$D_{Train}$
*and Train*
$CNN_{t1}^{1}$
*to obtain*
$H_{1}$*,**Save*$H_{1}$
   *End **for****Step 2: Concatenate new dataset from validation set prediction*
    *For*
i
*= 1, 2, …. m **do***

      $D_{h}=\left(x_{i}^{\prime },y_{i}\right)$*,*
   *End for**Step 3: Learn meta-classifier**Learn H based on the concatenated data****Output:** Ensemble classifier H*

### 2.1. Selection of Signal Processing Technique

As observed in the literature, signal processing is important in intelligent fault diagnosis of rotating machines. A careful selection/combination of the signal processing techniques can be beneficial to overcome the challenges posed by the different conditions to which rotating machinery are subjected.

For complementarity, cyclic spectral coherence, spectral kurtosis, and bicoherence analysis have been selected for this research. A combination of these techniques is expected to bring complementarity to expand the diagnostic sphere. Bearing defects produce modulating signals by their characteristic defect frequencies, which display second-order periodicity [21], that can be extracted or separated from other interfering signals and the hidden periodicity described. Cyclic spectral coherence is a robust diagnostic tool that can be used for this purpose in bearing and gear diagnosis. In the diagnosis of bearing in the presence of high impulsive noise, the cyclic spectral coherence gives good results [38]. However, in complex mechanical systems where the bearing signal produced is not strictly second-order cyclo-stationary, a series of non-zero values at non-fault related frequencies can occur. This can make the second-order cyclostationarity created by the bearing fault to be weakened [39].

The bicoherence analysis has also been prominent in determining the nonlinear interactions between frequency components in vibration data. Thus, making bicoherence analysis useful in rotating machine fault identification. It has been impressive by giving satisfactory results through random noise suppression ability in gear/bearing vibration data. Nonetheless, in some cases where quadratic phase coupling may not be present, the conventional bicoherence may still indicate peaks, thereby making it a little challenging to detect real nonlinear interactions between phased coupled components in the spectrum.

Spectral kurtosis provides a measure of the impulsivity of a signal. It has also been effective and widely used in the course of rotating machinery fault identification. Hence, spectral kurtosis can serve as a tool for selecting a band for frequency demodulation. However, in cases such as the multiple faults of shaft unbalance and bearing inner race fault, where the fault signal generates minor and major resonance frequency bands, spectral kurtosis may miss one of the frequency bands, thereby missing the faults in one of the components [40]. On the other hand, in gear faulted vibration signal interfered with high non-gaussian noise atthe machine’s natural frequencies, spectral kurtosis may encounter problems because the kurtosis value decreases as the transient’s recurrence rate increases.

#### 2.1.1. Cyclic Spectra Coherence

A stochastic process that displays hidden periodicity can be termed a cyclo-stationary process. A signal can be considered cyclo-stationary if it has periodic statistics. For a signal represented by *x*(*t*), to be considered a first-order cyclo-stationary signal with period *T*, it must produce a steady first-order moment which is periodic with period *T*. In a first-order cyclo-stationary signal, the mean or expected value is periodic, hence, Equation (1) holds.

(1) $$\;\;\;\;\;m_{x}\left(t\right)=m_{x}\left(t+T\right)$$

For the second-order cyclo-stationary signal in rotating machines, the autocorrelation function is periodic and can be described by Equation (2) below.

(2) $$R_{2x}\left(t,ꞇ\right)=R_{2x}\left(t+T,\;ꞇ\right)=E\left\{x\left(t-\frac{ꞇ}{2}\right)-x\left(t+\frac{ꞇ}{2}\right)*\right\}$$

where in Equation (2), * is the complex conjugate, ꞇ is the Time lag, *T* is the period and E[.] is the expectation operation. Hence, the cyclic autocorrelation function or the Fourier transform of the autocorrelation function can be estimated using Equation (3).

(3) $$\;\;\;\;\;R_{2x}\left(ꞇ,\alpha \right)=\int R\left(t,\;ꞇ\right)e^{-j2\pi \alpha t}dt$$

In Equation (3) α is the cyclic frequency. The Cyclic Spectral Correlation can be obtained by conducting Fourier transform on the cyclic autocorrelation function, and expressed as below in the first part of Equation (4) or as expressed in the second part of that same equation as the two-dimensional Fourier transform of the signal:

(4) $$\;\;\;Cr\left(\alpha ,f\right)=\;\;\int R\left(ꞇ,\alpha \right)e^{-j2\pi fꞇ}dꞇ=\iint R\left(t,\;ꞇ\right)e^{-j2\pi \left(\propto t+fꞇ\right)}dtdꞇ$$

Hence, the extent of correlation between two spectral components can be expressed with the Cyclic Spectral Coherence (CSCoh) through normalization of the Cyclic Spectral Correlation between 0 and 1 to obtain Equation (5).

(5) $$\;\;\;CSCoh\left(\alpha ,f\right)=\;\frac{Cr\left(\propto ,f\right)}{\sqrt{\left[C\left(0,f\right)\;\;C\left(0,f-\propto \right)\right]}}$$

#### 2.1.2. Spectral Kurtosis

Spectral kurtosis is a statistical parameter that indicates how the impulsivity of a vibration signal varies with frequency [41]. Dwyer described spectral kurtosis as the application of kurtosis in the recognition of hidden non-stationary components of a signal. Antoni [42] described spectral kurtosis by relying on the Wold–Cramer decomposition, which defines the output of a causal, linear, time-varying system as being a stochastic nonstationary process *X*(*t*) and given by Equation (6):

(6) $$X\left(t\right)=\int_{-\infty }^{+\infty }e^{j2\pi ft}H\left(t,f\right)dX\left(f\right)$$

In Equation (6), *dX*(*f*) is the associated spectral process, and *H*(*t*,*f*) is the time-varying function taken as the complex envelope of the process *X(t)* at a frequency *f*. The key assumption in which spectral kurtosis is applied is that the process must be conditionally non-stationary. It has been shown by previous researchers that a sizable number of conditionally non-stationary processes have the major property marked by a non-gaussian probability density function. Hence, the spectral kurtosis can be expressed as the energy normalizing fourth-order cumulant as:

(7) $$\;\;\;K_{x}\left(f\right)=\frac{\langle {\left|H\left(t,f\right)\right|}^{4}\rangle }{{\langle {\left|H\left(t,f\right)\right|}^{2}\rangle }^{2}}-2$$

In Equation (7), the temporal average operator is given by 〈.〉, and at frequency *f*, the complex envelope is described by H(t,f). However, for conditionally non-stationary processes having additive Gaussian noise, the spectral kurtosis is described by Equation (8) below:

(8) $$\;\;\;K_{x}\left(f\right)=\frac{K_{x}\left(f\right)}{{\left[1+\rho \left(f\right)\right]}^{2}}\;$$

#### 2.1.3. Bicoherence Analysis

The bicoherence analysis and bispectrum analysis belong to a class of analysis tools referred to as Higher Order Spectral analysis. The bispectrum analysis is a frequency domain measure that gives insight into the degree of coupling between three frequencies [43] *f*_1_, *f*_2_, and *f*_1_ + *f*_2_, which can be expressed mathematically as Equation (9).

(9) $$\;\;\;B\left(f_{1},f_{2}\right)=\;E\left[X\left(f_{1}\right)X\left(f_{2}\right)\;X^{*}\left(f_{3}+f_{2}\right)\right]$$

where *E*[.] is the expectation operation, the input vibration signal has a discrete Fourier Transform represented by *X*(*f*), and * is the complex conjugates. From Equation (9) above, it is observed that the bispectrum value is dependent on the amplitude of the Fourier transform [44] of $f_{1}$, $f_{2}$ and $f_{1}$ + $f_{2}$ and the coupling that exists between $f_{1}$, $f_{2}$ and $f_{1}$ + $f_{2}$.

The normalized form of the bispectrum gives the bicoherence spectrum. It helps to overcome the misrepresentation of the result by making the second-order properties of the vibration signal not to dominate the bispectrum. This normalized form of the bispectrum can be represented by Equation (10):

(10) $$b^{2}\left(f_{1},f_{2}\right)=\frac{{\left|B\left(f_{1},f_{2}\right)\right|}^{2}}{E\left[{\left|X\left(f_{1}\right)\;Y\left(f_{2}\right)\right|}^{2}\right]\;E\left[{\left|X(f_{1}+f_{2})\right|}^{2}\right]}$$

#### 2.1.4. Time Synchronous Averaging

Time Synchronous Averaging (*TSA*) is a signal processing method for removing periodic components from a noisy vibration signal [45]. The *TSA* is widely applied in gear vibration signal analysis. It is used to separate the vibration from the gear under investigation from noise sources and other gears that are not synchronous. That is, the *TSA* is used to average out the vibration signal over each revolution to eliminate the noise that is present [46]. Bechhoefer et al. [45] proposed an algorithm for implementing the *TSA*, which can be expressed through Equation (11).

(11) $$TSA=\frac{1}{M}\overset{M}{\underset{i}{\sum }}x_{i}$$

where *M* is the total number of samples, *i* is the resampled signal’s index, and $x_{i}$ is the resampled signal. To further improve the results obtained from the *TSA* signal, the residual signal can be estimated. This can be conducted by removing the gear mesh frequency, shaft frequency, and their harmonics from the *TSA* signal.

### 2.2. Convolutional Neural Network

**Figure 2:**
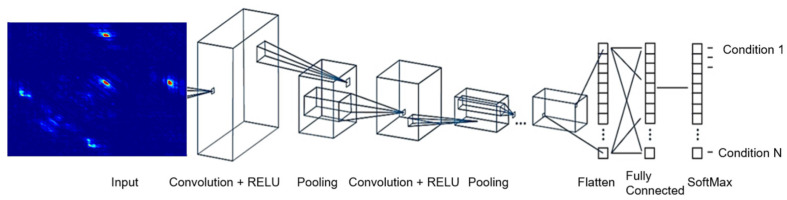
Convolutional neural network.

A convolutional neural network (CNN), as shown in Figure 2 below, is a supervised, feed-forward deep learning model designed for spatial hierarchical automatic learning of low-and-high-level features. CNN is biologically inspired and has been demonstrated to be effective in areas of image processing, video processing, natural language processing, and target tracking. CNN is made up of the input layer, convolutional layer, pooling layer, and fully connected layer.

A major layer in the CNN is the convolutional layer, where feature extraction is performed. The convolutional layer is made up of multiple learnable kernels, with each having a trainable weight and bias. It is at this layer that the convolutional operation occurs. Convolution is a mathematical operation in which convolutional kernels are applied to the input image to produce an output or feature map. Before the training of the CNN, the padding, strides, number of kernels, and size of the kernels are specified. A kernel is made up of a grid of discrete numbers or an array of numbers or values, with each value referred to as kernel weight [7]. Hence, the result at the end of the convolution becomes the input to the activation function to give the output of that convolutional layer.

A specific input image *X* to the CNN can be defined by a size *a x a x c*, where *a* is the height and width of the image, and the depth is given by *c*. The filter at the convolutional layer can be denoted by *k* and has dimensions *l x l x m*, with *l* being less than *a*, and *m* having a value that is less than or equal to c. With the weight given by *w*′ and the additive bias term given by *b*′, the features *h*′ are generated. The convolutional layer calculates the dot product between the input to this layer and the weights. By applying an activation function to these operations, the output of the convolutional layer is expressed using Equation (12) below.

(12) $$h^{\prime }=f\left(w^{\prime }*\;x+b^{\prime }\right)$$

where in Equation (12), *f* is the activation function. It can be of a varied type such as Rectified Linear Function or Logistic function or Tangent function.

The next operation in the CNN is the down-sampling operation which takes place at the pooling layer. Here, the number of learnable parameters is reduced. This operation helps to decrease the computational complexities of the model and reduces overfitting. Mathematically, the down-sampling or subsampling process is represented by Equation (13), where *β*′ is the multiplicative bias. A variety of pooling operations can be executed; this includes maximum pooling, gated pooling, and average pooling.

(13) $$X=f\left(\beta^{\prime }\;down\left(x\right)+b^{\prime }\right)$$

The final layer in the CNN is the fully connected layer. As the name implies, all the neurons in this layer are fully connected to the neurons of the preceding layer to create probabilities where different classes can be identified.

### 2.3. Blending Ensemble Learning

Stacked generalization is an ensemble learning method through which different base learners are trained simultaneously on the same dataset and a new trainable model based on *k*-fold cross-validation of the predictions of the base learners is used to obtain the final prediction of the model. Compared with a single model, the stacked generalization produces a model that has better bias-variance. However, stacked generalization comes with a baggage of being complex and becomes computationally expensive as the number of *k-*fold increases.

Blending is a variant of stacked generalization. In the blending ensemble learning approach, predictions from the base learners form inputs to the meta-learner without the use of *k-*fold cross-validation in generating this training data [47]. Thus, the burden of information leaks is reduced when compared with the stacked generalization. Despite deep blending ensemble’s requirement of high computation cost over single models, the absence of *k*-fold validation reduces this cost relative to stack generalization. The Error Correcting Output Code of MATLAB^®^ [48] is used as the meta-learner in this study. ECOC is a simple technique in which output features manipulation is performed through the solving of a subset of the given class problem [49]. That is, ECOC converts a multi-classification problem into a set of binary classification problems. A coding design is required by the ECOC, which controls the classes that the binary learners train on, and a decoding structure, which gives an estimation of how the predictions of the binary classifiers are made. Hence, in this architecture, the meta-learner is trained on the validation predictions of the base learners and tested on a new test set to obtain the final result.

## 3. Experiments

### 3.1. Experimental Description

The dataset consists of three parts comprising bearings, spur gear, and shaft. This is described in detail in the following subsections.

#### 3.1.1. GIDTEC Bearing Data

**Figure 3:**
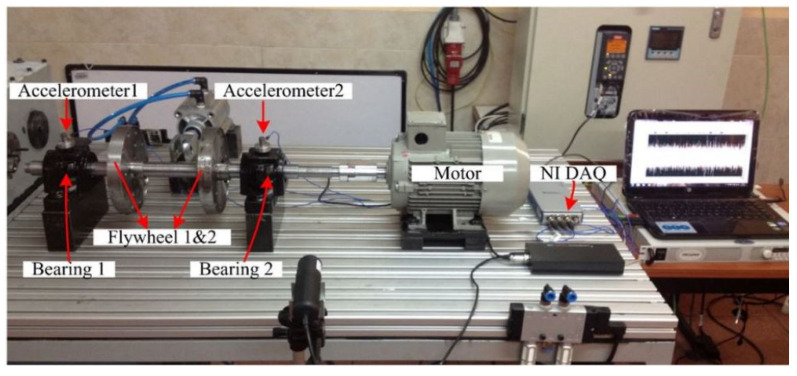
Experimental setup for the GIDTEC bearing dataset collection [50].

The experimental setup for the GIDTEC bearing data is shown in Figure 3. It is made up of two bearings with specification SKF 1207 Ektn9/C3 (bearing 1 and bearing 2) mounted in their housing, and two accelerometers (accelerometer 1 and accelerometer 2) that were stud mounted. The accelerometers have a sensitivity of 0.5 mV/g, and resonance frequency of ≥100 kHz.

The experimental setup was driven by an electric motor having a 30 mm diameter shaft on which the bearings are mounted. On this shaft, Flywheels 1 and 2 were mounted, from which no-load condition in the setup was denoted with L1, while loading condition two signified the application of Flywheel 2 on the setup and was denoted with L2. Flywheel 3 was used to introduce the loading condition L3. The experiment was repeated for run one to run five with the shaft rotating speed of 8 Hz, 10 Hz, and 15 Hz during each of the runs. Since bearing fault manifests in the high-frequency region, the bearing data with fault patterns indicated in Table 1 below were acquired at a sampling frequency of 50 kHz for 20 s. For this study, data collected with one sensor alone (accelerometer one) for six conditions were used, and it was channelled through the bearing data flow in Figure 1.

#### 3.1.2. Labelled Prognosis Health Management Society 09 Data

The labelled PHM09 dataset comprises six and eight conditions of the helical gear and the spur gear, respectively. However, for this work, the five conditions of the spur gear were utilized and are shown in Table 2 below.

**Figure 4:**
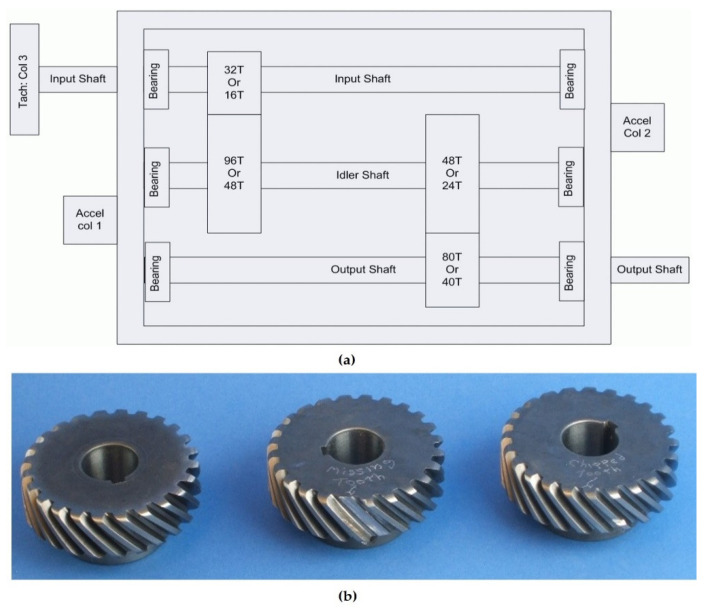
(**a**) Plan view of the experimental setup for the PHM09 dataset. (**b**) Healthy gear, gear with a missing tooth, and gear with a chipped tooth [51].

Originally, the dataset was acquired at a sampling frequency of 66.67 kHz for four seconds. Two accelerometers were stud mounted on the input and output shaft retaining plates. The accelerometers have a sensitivity of 10 mV/g, and resonance frequency > 45 kHz. The vibration signal from accelerometer one alone was used in this study. Additionally, attached to the experimental set-up was a tachometer that produces 10 pulses/revolution. The components of the gearbox included an input shaft, idler shaft, output shaft, bearings, and gears. The PHM09 data was collected at 30, 35, 40, 45, and 50 Hz shaft speeds, under high and low loading conditions. The number of teeth (T) on the spur gear mounted on the input shaft is 32 T. The idle shaft has two spur gears mounted on it, with the 96 T spur gear meshing with 32 T on the input shaft while spur gear 48 T meshes with spur gear 80 T mounted on the output shaft. A plan view of the gearbox is shown in Figure 4a, while Figure 4b indicates the image of some of the conditions of the gear used, for instance, healthy gear, missing tooth gear, and chipped tooth gear.

#### 3.1.3. COMFAULDA Data

The COMFAULDA data [52] were acquired with a sampling frequency of 50 kHz for five seconds. The conditions included normal, horizontal misalignment, vertical misalignment, unbalance, unbalance with horizontal misalignment, unbalance with vertical misalignment, and horizontal misalignment with vertical misalignment at various misalignment levels of 0.50, 0.51, 1.00, 1.27, 1.4, 1.50, 1.78, 1.91, and 2.00 mm. The experimental rig was operated under different shaft rotating speeds of 12, 13, 40, 50, and 60 Hz while subjected to loads of 6, 10, 15, 20, 25, 30, and 35 g at various times. The data was acquired using both stud mounted piezometric and capacitive accelerometer. However, for this research data, acquired from piezometric accelerometer having acceleration measurement range of −50 g to 50 g with 0.27 to 10,000 Hz frequency range, and sensitivity of about 100 mV/g [53], alone was used. The shaft data with their corresponding labels that were used include a healthy shaft (C2), a shaft having unbalance with vertical misalignment (C14), a shaft having unbalance with horizontal misalignment (C15), and a shaft having vertical misalignment with horizontal misalignment (C16). Hence, in all, sixteen single and multiple faulted shaft, bearing, and gear conditions from different machines were considered in this study.

### 3.2. Data Split

After performing the preprocessing on the datasets, this was divided into training, validation, and test sets using either different runs of the specific machine or different operating conditions of the machine. The training set and validation set are made up of 450 images having a size of 224 × 224 pixels obtained with colour scale adjustment based on amplitude content at the signal processing phase. As shown in Table 3, the dataset is small and will be susceptible to overfitting. A major cause of low generalization and poor accuracy of deep learning models is training on small data. This makes it difficult to build an effective model that produces reliable results. In this work, this challenge was resolved by using different data augmentation techniques. The bias in the position of the input images was resolved using vertical and horizontal translations of −5 and 5 pixels. The images which constituted the training/validation sets were safely rotated within the range of −15 to 15 degrees. These augmentation methods were implemented on the CSC-CNN, SK-CNN and BICO-CNN branches of the looped architecture.

Inputs of the preprocessed dataset using Spectral kurtosis, Cyclic Spectral Coherence, and Bicoherence, respectively, are fed to the base learners. Hence, SK-CNN represents a base learner on which Spectral Kurtosis maps or Kurtogram were given as the inputs. CSC-CNN signifies the base learner in which the Cyclic Spectral Coherence maps constituted the inputs. The BICO-CNN has inputs preprocessed using Bicoherence maps. The dynamics learning rate of the base learners was set at 0.001 for SK-CNN, 0.01 for CSC-CNN, and 0.001 for BICO-CNN. The topologies of the three base learners are given in Table 4 below.

An implementation of the ECOC involved training it on a concatenation of the predictions from the base learners obtained from the validation set and the validation class labels. The obtained ECOC model was tested using a concatenation of predictions obtained from base learners with the test set.

### 3.3. Description of Performance Metrics

The performance of the proposed method can be estimated using different performance metrics available. These performance metrics are hereby defined.

(a) Accuracy (*ACC*): Accuracy is one of the most widely used metrics to quantify the performance of machine learning or deep learning model. The accuracy of a given model provides the degree of effectiveness of the model. It is expressed mathematically by Equation (14) below, where *TP* is the True Positives, *TN* is the True Negatives, *FN* is the False Negatives, and *FP* is the False Positives:
  (14) $$\;\;\;\;\;ACC=\frac{TN+TP}{TP+FN+TN+FP}\times 100,$$
(b) Precision: It is the ratio of positive samples appropriately classified to the number of samples labelled as positive by the network. The precision of a deep learning model can be expressed as Equation (15):
  (15) $$\;\;Precision=\frac{TP}{TP+FP}\times 100,$$
(c) Recall: The recall of a model is an indication of the portion of the actual positive samples identified correctly. In other words, the recall gives the number of samples belonging to a class that was correctly classified. Mathematically, recall can be estimated using Equation (16).
  (16) $$Recall=\frac{TP}{TP+FN}\times 100,$$
(d) False Negative Rate (*FNR*): This metric is the probability that a true positive will be missed during a test by the network. The False Negative Rate is expressed mathematically by Equation (17):
  (17) $$FNR=\frac{FN}{TP+FP}\times 100,$$
(e) F1-score: This is the harmonic mean or average between the recall and the precision. This metric is expressed mathematically by Equation (18).
  (18) $$F1-Score=2\;x\frac{Recall\times Precision}{Reall+Precision}\times 100,$$
(f) False Positive Rate (*FPR*): The False Positive rate is the probability of samples predicted to belong to each of the classes that are incorrectly classified. It is also known as the false alarm rate and is given by Equation (19).
  (19) $$FPR=\frac{FP}{TN+FP}\times 100,$$

### 3.4. Signal Processing Optimization

In the proposed implementation of the architecture, the dataset to be fed into the network was preprocessed using the complementary techniques highlighted in Section 2 of this work. The dataset includes shaft raw data, a bearing dataset in which the DC component has been removed, and gear over rotations of the shaft to allow variable amounts of noise to be present before residual signal extraction.

Optimization of the signal processing techniques in this context refers to the tuning of the key parameters of the signal processing algorithm and training with a CNN model having the same hyperparameters to ascertain their performance. The steps in the optimization process include:

Step 1: A specified length of the signal is chosen for each of the data.
Step 2: Key parameters such as the decomposition level for spectral kurtosis (DL), the window length (*WL*) for cyclic spectral coherence, and the number of samples per segment for the bicoherence analysis are used in creating the kurtogram, cyclic spectral coherence maps, and bicoherence maps.
Step 3: Train the CNN using either the kurtogram or cyclic spectral coherence maps or bicoherence maps created with a specific key parameter.
Step 4: Repeat the CNN training with the next set of maps from the subsequent value of the key parameter and record the accuracy, precision, recall, and F1-score from each training.

In preprocessing with spectra kurtosis, using a specific signal length, various decomposition levels of 3, 6, 7, and 8 (for the bearing dataset) were used to obtain the kurtogram. It was noted that the maximum decomposition level $DL_{max}$ can be obtained from Equation (20) below, where “*x*” is the vibration signal. This is because, as the decomposition level increases, the plane between *f* and Δ*f* of the kurtogram becomes finer for narrow-band detection [22].

(20) $$DL_{max}=Log_{2}\left(Signal\;lenght\left(x\right)\right)-7,$$

**Figure 5:**
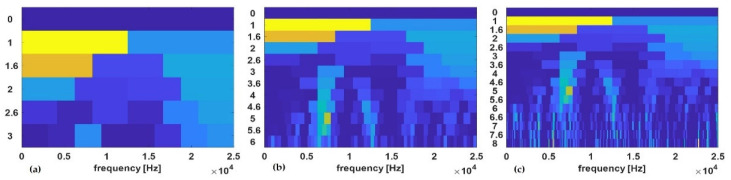
Kurtogram of healthy bearing at: (**a**) Decomposition Level 3, (**b**) Decomposition Level 6, and (**c**) Decomposition Level 8.

**Figure 6:**
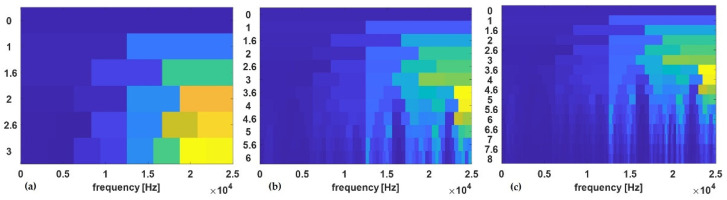
Kurtogram of inner race fault in bearing 1, ball fault in bearing 2: (**a**) Decomposition Level 3, (**b**) Decomposition Level 6, and (**c**) Decomposition Level 8.

Figure 5a–c, presents the kurtogram at all considered decomposition levels for the healthy bearing. Here, the healthy bearing from run 1 of the machines, with a rotating speed of 8 Hz and no load, L1, was considered. Figure 6a–c, shows the considered decomposition level for condition label *C6* having an inner race fault in bearing 1, and a ball fault in bearing 2. It can be observed that narrower bands can be detected in Figure 5c and Figure 6c, unlike Figure 5a and Figure 6a.

Similarly, for the Cyclic Spectral Coherence maps, different window lengths/sizes were used. The magnitude of the length of the window (*WL*) chosen affects the frequency resolution (Δ*f*) as indicated by Equation (21) below. Where *Fs* is the sampling frequency.

(21) $$\Delta f\;∼\;\frac{Fs}{WL},$$

**Figure 7:**
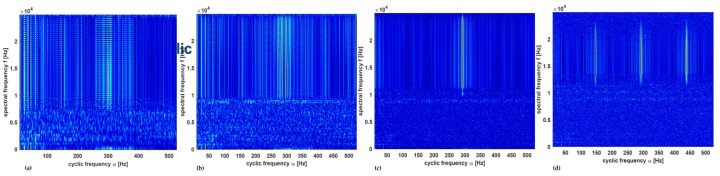
Cyclic spectral coherence maps for healthy shaft at window size: (**a**) 128, (**b**) 256, (**c**) 512, and (**d**) 1024.

Thus, a large value of the window length would result in better performance of the CSC-CNN due to the impact of higher frequency resolution. The elapsed time (computational time) is also influenced by the length of the window for a chosen length of the signal used for the computation of the cyclic spectral coherence maps. For optimization with the cyclic spectral coherence, window lengths of 128, 256, 512, and 1024 were used in creating cyclic spectral coherence maps on a signal length of 40,080 data points of the shaft data. Figure 7a–c, shows the cyclic spectral coherence maps for the healthy shaft having condition label *C2*. Performance metrics such as overall test accuracy, precision, recall, and F1-score of the trained CNN model using input maps from a specific window length were recorded.

**Figure 8:**
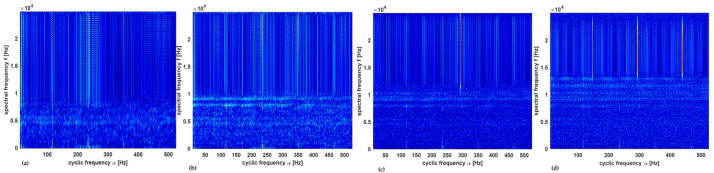
Cyclic spectral coherence maps for unbalance shaft at window size: (**a**) 128, (**b**) 256, (**c**) 512, and (**d**) 1024.

The signal with condition label *C6* was used in creating the cyclic spectral coherence maps with different window sizes in Figure 8a–c. Through inspection, it was detected that for this signal length, as the window size decreases, the cyclic spectral coherence maps became more coarse.

**Figure 9:**
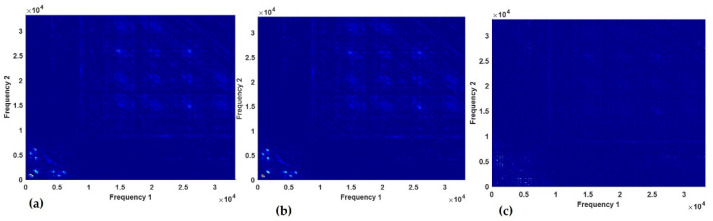
Bicoherence maps for healthy gear with the number of samples per segment as: (**a**) 256, (**b**) 260, and (**c**) 1024.

In the optimization of bicoherence maps, a key parameter that was varied was the number of data samples per segment. Different data sample sizes were used such as 256, 260, and 1024 with the corresponding Number of Fast Fourier Transform lengths (NFFT) of 256, 512, and 1024, respectively. Figure 9a–c shows the bicoherence maps of the healthy spur gear with the condition label of *C3*.

**Figure 10:**
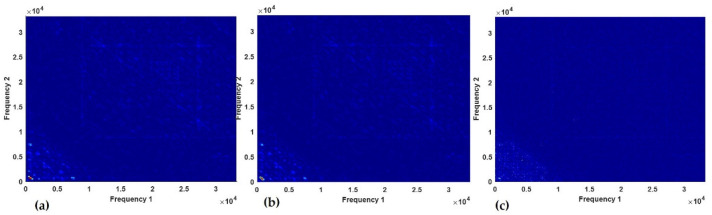
Bicoherence maps for C10 with the number of samples per segment as: (**a**) 256, (**b**) 260, and (**c**) 1024.

The damaged spur gear with condition label *C10* has fault conditions of a chipped tooth and eccentric gear in the signal. This is indicated in the bicoherence plots of Figure 10a–c below. The bicoherence maps show that the quadric phase coupling between the interacting components was more visible in the plot with the number of samples per segment being 260.

## 4. Results and Discussions

### 4.1. Optimization Results

**Figure 11:**
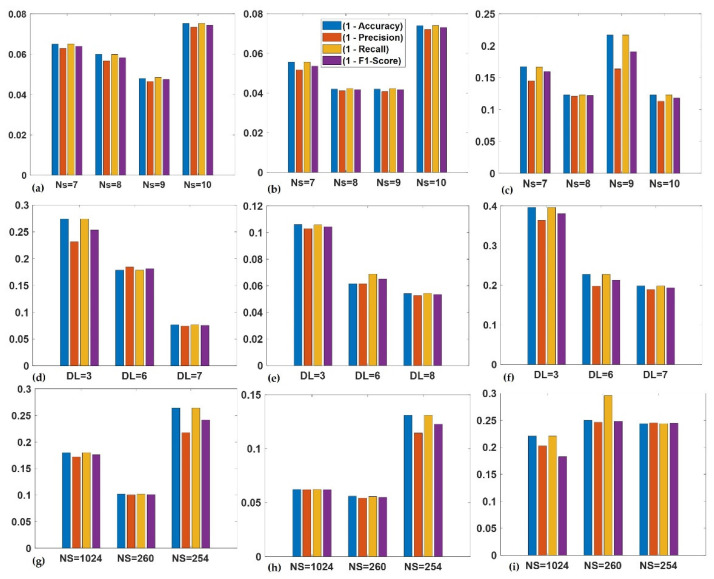
Optimization of model based on (**a**) CSC of shaft, (**b**) CSC of bearings, (**c**) CSC of gear, (**d**) SK of shaft, (**e**) SK of bearings, (**f**) SK of gear, (**g**) bicoherence of shaft, (**h**) bicoherence of bearing, and (**i**) bicoherence of gear.

The images from the different key parameters of spectral kurtosis, cyclic spectral coherence, and bicoherence formed input to thirty (30) CNN models with training conducted using a common CNN topology and hyperparameter. The overall accuracy, overall precision, F1 score, and overall recall were the metrics used to quantify the performance of the models. However, in Figure 11, one minus each of these metrics (known here as error) was plotted to analyse the performance of these models. Across the shaft, bearing, and gear spectral kurtosis-based CNN models, it is observed that decomposition level three displayed a test accuracy error of approximately 0.05 for the shaft at the decomposition level of 7 and about 0.4 for the gear at the decomposition level of 3. Similarly, the error due to the overall precision was highest in the gear model at approximately 0.35 at a decomposition level of 3 and about 0.1 for the shaft model with a decomposition level of 7 as shown in Figure 11d–f. In summary, it can be noticed that among the three machines, the error in the test accuracy reduced from decomposition level 3 to decomposition level 8. As a result, the selected spectral kurtosis decomposition level for the integrated model for shaft, gear, and bearing data were 7, 7, and 8, respectively.

The results presented in Figure 11a–c were obtained from the training of the models with cyclic spectral coherence using window sizes ranging from 128 to 1024. It can be noticed that the error due to the overall recall was about 0.04 for the shaft and bearing data at a window length of 512. The value of the recall increased at a window length of 128 to a maximum at a window length of 512. The recall decreased to 0.9247 for the shaft cyclic spectral coherence model as shown in Figure 11a. For the gear dataset, the overall recall was highest with a window length of 256. Hence, in this study, a window size of 512/512 and 256 for the bearing/shaft and gear datasets, respectively, were used for making the cyclic spectral coherence maps.

The bicoherence maps inputs with the number of samples per segment as 260 and NFFT of 512 gave the best performance throughout the various bearing and shaft models. Hence, as shown in Figure 11g,h, one minus *F-1* score value that is less than 0.1 was obtained for these models. In Figure 11i, the bicoherence model with inputs obtained from 1024 samples per segment produced the best performance for the gear data. Since this parameter produced the best performance and the difference between the elapsed time for other segment sizes for each map for the gear was less than 2 s, the 1024 samples per segment was chosen.

### 4.2. Testing

**Figure 12:**
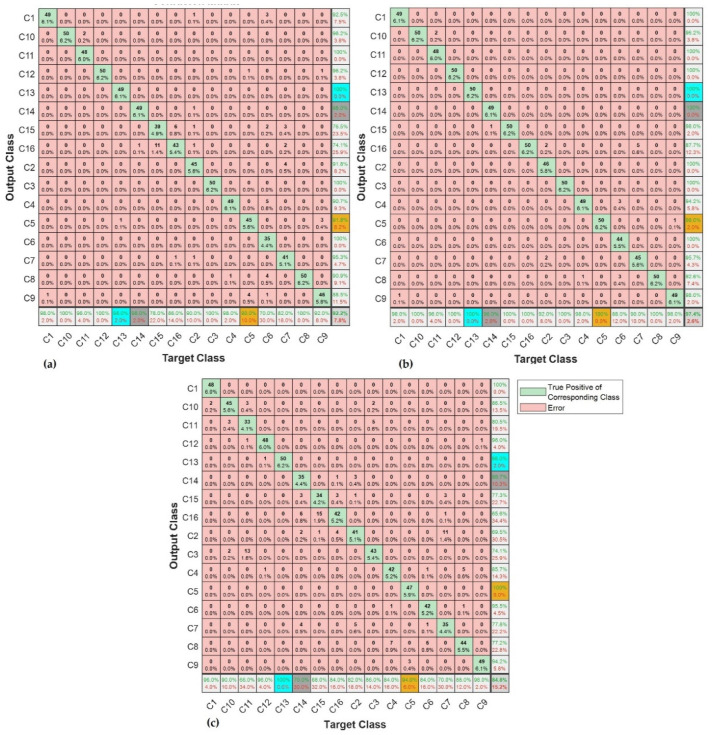
Performance of: (**a**) SK-CNN; (**b**) CSC-CNN; and (**c**) BICO-CNN.

The performance of the base learners was quantified separately using the metrics previously defined in Section 3 of this article. The test in this context was implemented using the test data shown in Table 3. The results from base learners are presented through the confusion matrixes shown in Figure 12a–c.

In all the base learners, SK-CNN returned an overall test accuracy of 93.29%. The overall precision and overall recall for this learner were 0.9266 and 0.9239, respectively. SK-CNN also produced an F1-score of 0.9252. CSC-CNN, which had input from the cyclic spectral coherence maps, presented an overall test effectiveness of 97.38%. The overall recall and precision of this learner were 0.9738 and 0.9753, respectively. The bicoherence input base learner showed an overall test accuracy of 84.75%. BICO-CNN also presented an overall precision of 0.8851 and an overall recall of 0.8776. Based on the overall test accuracies, it can be observed that the CSC-CNN base learner performed better than the SK-CNN, and BICO-CNN models.

In Figure 12, the row at the bottom of each of the confusion matrixes shows the true positive rate in green font and the false negative rates in red font. An indication of correctly classified samples for each class or condition of the machine by the specified base learner is shown in the diagonal of the confusion matrix. For instance, in Figure 12b, in the multiple faults shaft signal with condition label *C14*, 49 examples from a total test set of 800 were accurately classified to belong to this class by CSC-CNN. The other base learners displayed a performance where 49 and 35 examples from the entire test set were correctly classified as *C14* by SK-CNN and BICO-CNN, respectively. This constituted 6.1% of the total test set used in this case for SK-CNN as well as CSC-CNN, and 4.4% of the total test set for the BICO-CNN. The last column in each of the confusion matrixes shows the false positive rate in red and the precision in green. Alternatively, the last row of the confusion matrix shows the recall and the false negative rate. Hence, the SK-CNN indicated a precision of 98.0%, a recall of 98.0% for condition label *C14*, and BICO-CNN presented a slightly high false positive rate of 10.3% for *C14*. The CSC-CNN indicated a perfect performance for the *C14* fault condition. This is shown with the face colour grey in the confusion matrixes. It returned a precision of 100.0%, and a false positive rate of 0%. However, it also returned a recall of 98.0% with a false negative rate of 2.0%. These indices show that the results for the shaft fault condition *C14* of the three base learners were complementary, with the CSC-CNN base learner showing a superior performance to the SK-CNN.

Further complementarity amongst the base learners can be demonstrated through closer consideration of their performance using the confusion matrixes in Figure 12a–c. Taking the bearing fault *C5* having a face colour of orange, where the signal is made of an outer race fault in bearing 1, healthy bearing 2; the recall for BICO-CNN was 94.0%, 90.0% for the SK-CNN, and 100% for CSC-CNN. The precision and the false positive rate were 100.0%, and 0% for BICO-CNN. SK-CNN presented a precision of 91.8% and a false positive rate of 8.2%. With the CSC-CNN model, these metrics were 98.0% and 2.0%. This means that for that bearing multiple fault conditions, the BICO-CNN was the best precision indicator and was strongly supported by the CSC-CNN model.

Considering the fault with condition label *C13* having a face colour of blue in Figure 12a–c, where there are gears chipped tooth on gear 32 T, eccentric gear on gear 48 T, and broken tooth on gear 80 T in the signal, the cyclic spectral coherence base learner still outperformed the other base learners with a precision and recall of 100%, and a false alarm rate and missed detection rate of 0%. However, the bicoherence base learner presented a solution with a recall of 100%, and a precision of 98.0% for this condition. While the SK-CNN indicated 100% precision and recall of 98.0%. From the foregoing, it can be observed that for this multiple-fault gear condition, the recall of the spectral kurtosis base model was the same as the precision of the BICO-CNN base learner. Thus, reflecting a measure of complementarity between the base learners for this rotating machine component’s condition.

**Figure 13:**
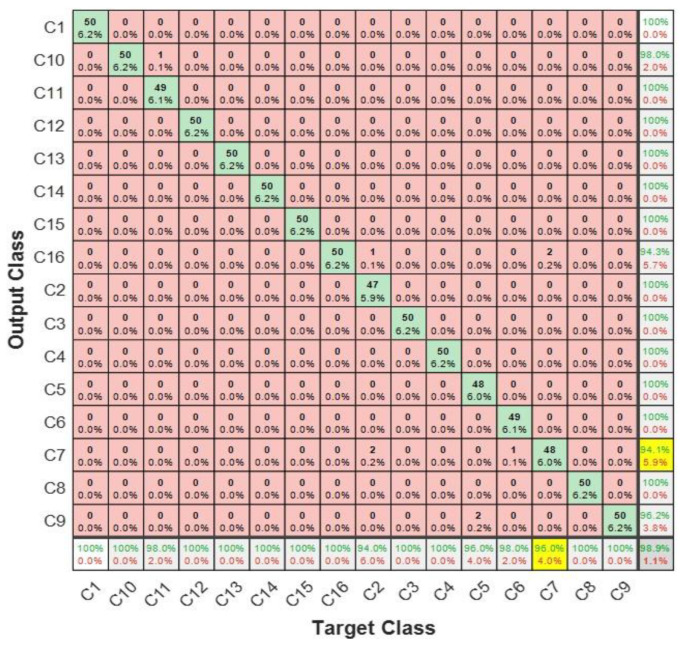
Confusion matrix for the proposed ensemble model implementation.

The ECOC was used as the meta-learner in the proposed method. The predictions from the base learners were concatenated into an ‘*N* × *M*’ matrix, which formed input to the meta-learner. Where ‘*N*’ is the number of observations or samples, and ‘*M*’ is the total number of classes on which the meta-learner was trained before testing. The confusion matrix in Figure 13 shows the overall test accuracy of the proposed diagnostic model to be 98.9% with an overall precision and overall recall of 0.9892 and 0.9888, respectively. The model also presented an F1 score of 0.9890. It was also observed that the unbalance shaft fault having a condition label *C7* with the face colour yellow returned the highest false positive rate of 5.9%. This condition of the machine also showed the highest false negative rate of 4.0% in Figure 13. However, this was lower than that of any of the base learners for that condition.

To study how this ensemble learning implementation has addressed the issues in the base learners across the different rotating machine components using representative cases, the bearing signal with condition label *C1* showed a recall/precision of 100% for the ensembled model. This was an improvement of 2% and 4% in the recall for the SK-CNN/CSC-CNN, and BICO-CNN, respectively. For the multi-fault gear signal with condition label *C10*, the precision performed better by 1.8% when compared to the best precision for this condition from other base learners. A 2% increase in recall was noticed for the ensemble learning model over the CSC learner for condition *C11*. However, for the component with condition labels *C12* and *C13*, the proposed implementation returned 100% for recall and precision. This equalled the performance of the best base learner: CSC-CNN. While the missed detection rate for the shaft fault signal having unbalance with vertical misalignment, *C14*, was 2.0% in the spectral kurtosis and cyclic spectral coherence learners, our ensemble model, eliminated this occurrence. For condition label *C15*, the false alarm rate, which was present in all the base learners at various degrees, was also eliminated by the proposed method.

In this study, the weakest bearing fault signal was the signal with condition label C4 with SNR of −25.15 db. CSC-CNN returned a false positive of 0.02 and a false positive rate of 0.058. This was 3.5% better than the false positive rate of SK-CNN. Hence, the established complementarity in the proposed architecture resulted in best performance with this fault condition. Thus, the precision as well as the recall for the condition was 100%, with 0% miss detection rate and fault alarm rate.

### 4.3. Feature Analysis

**Figure 14:**
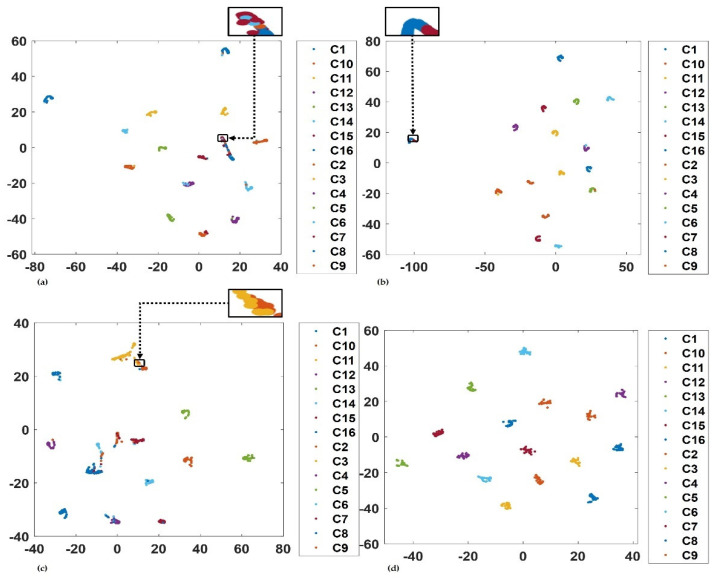
T-SNE representation for the last layer of the base learner: (**a**) SK-CNN, (**b**) CSC-CNN, (**c**) BICO-CNN, and (**d**) proposed method.

To further demonstrate complementarity in the architecture aside from the selection of complementary signal processing technique explained in the previous section, the t-Distributed Stochastic Neighbour Embedding (t-SNE) technique is used in this article to visualize the features in the base learners using the test data. The t-SNE was developed by Maaten et al. [54] for visualization. It maps high-dimensional data to two-dimensional space by using nonlinear maps that preserve distance. Figure 14a–d shows the t-SNE of all the base learners and the proposed implemented model. Sixteen colours that represent the conditions of the machines are used to indicate and group the distinctive features from the test dataset. Ideally, features from the same condition of the machine are clustered together.

However, it is observed from the t-SNE distributions that there was an overlap between features using test data in the different base learners. For instance, in the SK-CNN in Figure 14a, there was a prominent overlap between observations of conditions *C15* and *C16*. An exaggerated view of this overlap is shown in the top right corner of the same figure. This indicates that the features were not so discriminative. The CSC-CNN in Figure 14b, presented a better discriminative performance. The amount of overlap between the feature observations from each of the groups was reduced and different from that of SK-CNN.

Comparably, an inspection of Figure 14b, also shows that an overlap occurred between some observations in *C9* and *C5*. The BICO-CNN base learner returned a cluster of features. The software tools indicated a visible overlap between observations in groups *C3* and *C10* of the features in Figure 14c. However, the t-SNE distribution for the proposed implemented model using the test data, returned an excellent discrimination between the features. Hence, in Figure 14d, all the observations are separated, without any visible overlap between the observations.

### 4.4. Comparison with Other Deep Ensemble Methods

Some scholars have performed compound fault and multiple faults diagnosis of the rotating machine previously using different deep ensemble learning models. Table 5 shows a comparison of the overall accuracy or effectiveness of the proposed model implementation and other state-of-the-art methods. It indicates an improved performance from the proposed implemented model, and also shows that there was an extended coverage of multiple faults across three rotating machine parts for the proposed method.

## 5. Conclusions

In this study, an integrated architecture for multiple faults diagnosis across different rotating machine components was implemented. Optimized complementary signal processing maps obtained through the selection of optimal parameters for bicoherence, cyclic spectral coherence, and spectral kurtosis within the deployed signal length were inputted into the framework for fault diagnosis. The maximum decomposition level for specific signal length in the use case of spectral kurtosis gave the best performance with the learners. The bicoherence learner with 260 samples per segment in our case, gave good effectiveness for the optimized models across the shaft and bearing rotating components. The elapse time was more for this learner when it has a higher number of samples per segment. Thus, while a higher choice of samples per segment for a branch may not guarantee improved performance, it also results in more computational burden. The cyclic spectral coherence input optimization produced superior overall effectiveness at a window size of 512 for the bearing/shaft dataset, although lower for the gear dataset.

The results from the proposed model showed improved performance across different rotating machine components of bearing, gear, and shaft when tested with a dataset of varied operating conditions. An improvement of 1.5% in the overall accuracy of the blending ensemble learning approach was recorded over the result from the best-performing individual classifiers. The mean overall accuracy of the individual classifiers showed that our implementation presented a 7.43% increase in mean overall effectiveness. Specifically, for the weak fault with condition label C4, the risk of missed detection and false alarm were significantly reduced through the proposed model in comparison to the individual learners. Compared with other state-of-the-art ensemble approaches for multiple faults of rotating machines, our proposition presented improved mean overall effectiveness of 4.47%. Although optimization of the signal processing techniques was carried out, future work will study the effect of the signal processing parameters on image input size, establishing a control group without any signal noise reduction treatment to verify its significance, and evaluation of the performance of the proposed system to industrial setting.

## Figures and Tables

**Table 1:** Bearing conditions.

S/No.	Label	Bearing 1	Bearing 2
1	C1	Healthy	Healthy
2	C4	Inner race fault	Healthy
3	C5	Outer race fault	Healthy
4	C8	Inner race fault	Outer race fault
5	C6	Inner race fault	Ball fault
6	C9	Outer race fault	Ball fault

**Table 2:** Gear conditions.

Gear				Bearing						Shaft	
Label	32 T	96 T	48 T	80 T	IS: IS	ID: IS	OS: IS	IS: OS	ID: OS	OS: OS	Input
C3	Good	Good	Good	Good	Good	Good	Good	Good	Good	Good	Good
C10	Chipped	Good	Eccentric	Good	Good	Good	Good	Good	Good	Good	Good
C11	Good	Good	Eccentric	Good	Good	Good	Good	Good	Good	Good	Good
C12	Good	Good	Eccentric	Broken	Ball	Good	Good	Good	Good	Good	Good
C13	Chipped	Good	Eccentric	Broken	Inner	Ball	Outer	Good	Good	Good	Good

**Table 3:** Composition of training, validation, and test datasets.

Component	Training Set, Images	Validation Set, Images	Testing Set, Images
Bearing	400	50	50
Gear	400	50	50
Shaft	400	50	50

**Table 4:** Topology of the base models.

Layer	Description	SK-CNN	CSC-CNN	BICO-CNN
1	Input	224 × 224 × 3	224 × 224 × 3	224 × 224 × 3
2	conv_1	8 × 5 × 5 × 3	8 × 5 × 5 × 3	8 × 5 × 5 × 3
3	maxpool_1	3 × 3	3 × 3	3 × 3
4	conv_2	16 × 5 × 5 × 8	16 × 5 × 5 × 8	16 × 5 × 5 × 8
5	maxpool_2	3 × 3	3 × 3	3 × 3
6	conv_3	32 × 5 × 5 × 16	32 × 5 × 5 × 16	32 × 5 × 5 × 16
7	maxpool_3	3 × 3	3 × 3	3 × 3
8	conv_4	64 × 5 × 5 × 32	64 × 5 × 5 × 32	64 × 5 × 5 × 32
9	maxpool_4	3 × 3	3 × 3	3 × 3
10	conv_5	128 × 5 × 5 × 64	128 × 5 × 5 × 64	128 × 5 × 5 × 64
11	dropout	70%	70%	70%
12	fully Connected	fully Connected	fully Connected	fully Connected
13	SoftMax	1	1	1
14	class output	16	16	16

**Table 5:** Comparison of the proposed approach with other ensemble deep learning methods.

S/N	Reference	Method	Faults Considered	Accuracy (%)
1	Ma et al. [33]	CNN, Autoencoder, and Deep Belief Network Ensemble	Rotor and bearing single fault	98.09
2	Sr et al. [55]	Decision fusion of CNN and MLP	Gearbox mixed signal	96.07 (Ave)
3	Inyang et al. [56]	CNN-SVM	Bearings	98.54
4	Martins et al. [53]	Random Forest	Compound fault in rotating machinery	81.41
5	Zhang et al. [57]	Ensemble Deep Contractive Autoencoder	Gearbox and Bearings	96.60 (Ave)
6	Li et al. [58]	Ensemble Deep learning with Beetle Antenna Search-	Rail locomotive bearings	96.92
7	Han et al. [59]	Dynamic Ensemble Convolutional Neural Network	Gearbox	96.48 91.30
9	Proposed	Proposed	Bearing, Gear, and Shaft Multiple faults	98.9

## Data Availability

Not applicable.
